# Foreign

**Published:** 1841-06-12

**Authors:** 


### FOREIGN.

*History of a Remarkable Case of Phlebitis; with observations.* By THOMAS HOOKHAM SILVESTER, M. D., Member of the College of Physicians, and Physician to the South London Dispensary.—Much difference of opinion exists in regard to the treatment of phlebitis, and much remains to be known respecting its cause, nature, and consequences. All are agreed as to its danger. It is the object of the author of the paper to describe the disease, its phenomena during life, and the pathological appearances after death, as they occurred in an isolated case. The patient, about sixty years of age, the subject of piles and large varicose veins in both extremities, received a slight wound, probably from his razor, in the upper lip, which was followed by enormous swelling of the part, but not much constitutional disturbance. At the end of fourteen days the disease seemed to have finished its course, and the patient to be recovering. It quickly, however, reappeared in the veins on each side of the nose, and progressively extended through the numerous ramifications of the frontal and temporal vessels, which, on opening with a lancet, poured forth in abundance "laudable" pus. Incrustations something like the scabs of rupia, appeared along the track of each vessel, and when these were removed the interior of the vein became exposed, and the healing process went on by granulation. The symptoms of the disease were, from the commencement to the termination, of a very mild character. The patient appeared to sink under exhaustion at the end of the ninth week, at a moment when pus existed only in a few of the vessels about the vertex; the process of reparation having been completed in the lips, sides of the nose, and forehead. The autopsy discovered pus in the trunks, and a fibrinous crumbling substance in the ramifications of part of the venous system of the scalp. The minuter branches contained a little fluid blood, of which, however, there was a very small quantity in the whole body. No pus globules could be traced by the microscope. Deposits of pus were sought for in the several large organs and in the muscles, but fruitlessly.

Dr. James Johnson said it would be highly interesting to hear the opinions of the many able surgeons then present, respecting the mode of treatment best to be adopted in the early stages of phlebitis; whether, he particularly meant, the antiphlogistic or the tonic plan of treatment should be followed.

The President said the question was one to which it was impossible to give a definite answer. Phlebitis occurred under such a variety of different circumstances, and put on so many different characters, that it was impossible to lay down any general rule of treatment. The cases of phlebitis, for example, which were attended with great enlargement of the leg, and passed by the name of phlegmasia dolens, were almost always quite chronic in their course, and required no active treatment at all. Other cases, on the contrary, were most rapid and acute: he had known them terminate fatally in three or four days from the commencement of the symptoms; and one case he had seen had proved fatal in only forty-eight hours. He could give no general statement for the ordinary cases of phlebitis, but, as far as his experience went, he should say that general bleeding was rarely useful: the condition of the patient was more commonly such as to require support.

Mr. Skey said, that his experience led him to regard the antiphlogistic treatment of phlebitis as very rarely beneficial, and often injurious. In the practice at St. Bartholomew's Hospital he had had opportunities of observing how very frequently the disease supervened in those who were of a weak debilitated state of system, and more especially in those who had suffered from great loss of blood. He remembered in particular one case, of a man, who was bled there to 120 ounces in the course of an hour, for the reduction of a dislocation of the hip; about 25 grs. of tartar emetic were given to the man in the same time: and at the time he (Mr. S.) expressed his conviction to a friend standing by, that the patient would die of phlebitis. He anticipated this partly from the quantity of blood lost, and partly from the more than ordinary violence which it was necessary to employ, in order to obtain so considerable a quantity of blood from the arm. The hip was happily reduced, and the man was put to bed apparently well; but phlebitis came on, as he had anticipated, and the man died. He remembered another case very similar to this, in which, however, the quantity of blood abstracted was less, and no unusual injury was inflicted on the vein; but in this also phlebitis ensued, and ended fatally. From these cases, and from the general results of his experience, he believed that bleeding should not be employed in cases of phlebitis.

Mr. Dalrymple related a case in some measure confirmatory of the same view, which was still partially under his care. It was that of a medical student, who had reduced himself to a state of great weakness by the abstinence and energy with which he pursued his studies: eating meat only three times a week, that it might not prevent his application, and sitting up reading till two or three o'clock in the morning. In the condition which these habits, united with an originally very irritable constitution, brought on, he had received a slight injury; he had scratched and irritated a slight eczematous eruption in his leg, and it was followed by swelling in the groin, and phlebitis of the veins of the leg and thigh. He was, under the care of a physician, treated by severe antiphlogistic measures: his diet was reduced to the lowest quantity, and leeches repeatedly applied, and reducing measures were administered. By these means he was soon brought to the lowest possible stage of debility; and at length, from the typhoid state which came on, it was found absolutely necessary to administer cordials, and to support him. A deep abscess now formed on the inner side of the thigh, which was opened, the incision being extended through the fascia, and a quantity of matter discharged; he now seemed going on well, but some days afterwards hæmorrhage to a considerable extent took place from the opening into the abscess. The signs of phlebitis now again returned, and fresh abscesses formed in the course of the veins of the leg. The hæmorrhage was removed at several times, but was checked by pressure; on one day, however, he suddenly lost nearly a quart of blood in a very short time. Mr. Liston and Mr. Travers being then called in, it was determined to tie the femoral artery; and this being done, the hæmorrhage did not again return, and the patient again began to rally and to make progress. He went on well till one day he exerted himself too much, and moved his leg about, when again phlebitis came on, and only a few days ago a large abscess in the thigh had burst. Now, however, he seemed in so much more favourable a state, that recovery might fairly be anticipated. The whole course of the disease, however, in th s case seemed to indicate clearly that reducing measures were injurious; and he could not but think that had the treatment been from the commencement more calculated to support the strength of the patient, the result would have been much more satisfactory.

Mr. Arnott said he could not agree in the condemnation of antiphlogistic measures, and especially of the abstraction of blood, for he held that, in a certain form, that measure was the most advantageous that could be employed; and indeed, in his opinion, the only one of real benefit. Much had been said of the tendency of phlebitis to occur in the debilitated and reduced, and especially in those who had suffered from large losses of blood. Such cases, no doubt, did occur; but they did not constitute the majority of cases. He conceived that it must be familiar to the members that, after amputation, phlebitis is much more common in the cases in which the operation is performed directly after an accident in robust and previously healthy persons, than in those who had been previously exhausted by long-continued scrofulous or other disease. He had seen numerous proofs of this, but he would mention only one: a lad, 15 years old, was brought to the hospital, having had his leg crushed by a railway engine: within three hours of the accident he amputated the thigh, and found the femoral vein already blocked up by coagulum. He expressed at once his assurance that the boy would have phlebitis; and he had, and died. The vein had, no doubt, received a severe injury by the accident, and inflammation had at once commenced in it. Now it was contrary to all that had ever been taught, and to what daily experience constantly confirmed, to imagine that cases of this kind should be benefited by any thing but antiphlogistic treatment: but he would make a distinction as to the kind of bleeding to be employed. He did not believe that bleeding from the arm was generally useful: the plan to be followed was to apply leeches again and again over the inflamed veins: to keep the blood constantly flowing from around them. And with this mode of treatment the administration of nourishment was not, in cases where it was deemed necessary, at all incompatible: the two measures might be employed together without any impropriety.

With respect to the case now before the Society, he regretted the author was not present, that he might have asked for explanation of several points connected with it. He confessed that he had doubts whether it were a case of phlebitis at all; it had rather the characters of inflammation of the lymphatics; and he should like to know whether any of the collections of pus had been fairly traced to have connection with the veins of the scalp.

Dr. Williams said he was present at a part of the examination of the body, and he could state certainly that the deposits of lymph and pus were traced into the veins. The question raised to-night with reference to the connection between a reduced state of the system and inflammation of the veins, was one of great interest. He was not aware that in medicine there was any class of cases comparable with these which fell under the care of the surgeon; unless, indeed, it were those of dropsy from diseased kidneys, with albuminous urine. In these, in which it was well known that the blood becomes greatly impoverished, he had of late noticed a remarkable tendency to the occurrence of disease of the arteries and their valves. In the last six cases observed at St. Thomas's, this had been the case; it seemed certain that before the dropsy occurred, and keeping pace with the impoverishing of the blood, extensive disease had been going on in the large arteries and the aortic valves. The author had alluded to his having regarded this case, in the first instance, as one of ordinary erysipelas: he (Dr. W.) thought the connection of the two diseases a subject well worthy of inquiry. He believed that it was held by M. Velpeau that phlebitis is very frequently coincident with erysipelas.

The President said there was no doubt of the fact; he had often examined cases of erysipelas, in which, with sloughing of the subcutaneous cellular tissue, the veins were highly inflamed. With reference to the occurrence of phlebitis in persons in previously good health, there could be no doubt that it was a common occurrence. When he was a student at St. George's Hospital, it was the custom to tie the vena saphena for varicose veins of the legs, and cases of phlebitis were then very common. The operation was discontinued partly because it did no good; for the patients used to come back three or four months afterwards with their legs as bad as ever; and he believed that all the good it did for the short time was only owing to their having been kept in bed. But the chief reason for giving it up was, that many of the patients had severe phlebitis after the operation, and died. Now these were all people in previously perfectly good general health. In many of these he remembered blood-letting was employed to a considerable extent, and it seemed to produce benefit in some instances. He had himself, when assistant surgeon, at the suggestion of Mr. Abernethy, divided the saphena vein in a healthy man for a varicose state of the veins of the leg. In this case phlebitis of great severity came on, and the man was largely bled, and recovered.

Mr. Arnott said he had also had several occasions of examining cases of erysipelas, in which he had found both the veins and the lymphatics of the part highly inflamed and filled with pus. The case related by Dr. Sylvester, he must still remark, differed widely from ordinary cases of phlebitis. In the first place, its origin was different; it was not usual for phlebitis to be produced by the irritation of a pimple, or to commence with a slight local inflammation: but this was very commonly the case with inflammation of the lymphatics. In the second place, the course of the disease was different; phlebitis almost always proceeded along the large trunks of the veins; this had gone through a number of branches: and, lastly, the character of the matter discharged was different; it was described as pure laudable pus; but that of phlebitis was ordinarily, not perhaps always, but still generally, of a reddish colour, and sanious.

Dr. Ashwell said he thought that in relation to the question of bleeding, a distinction ought to be made between cases that occur from any accidental injury, or from any other traumatic cause, and those that come on as if spontaneously. In the former class he had no doubt blood-letting was highly beneficial; but, in other cases, in those, for example, which occurred after a severe labour, he had never seen it of any value.

Mr. Macilwain said it appeared to him that there was a certain character of constitution common to all cases of phlebitis, with which it was necessary to be acquainted before it could be determined what treatment was adapted to it. It was difficult, perhaps impossible, to define exactly what character this was; but as far as he had observed, it was always marked by a want of power. He should, therefore, not be inclined to adopt active antiphlogistic measures; and he almost regretted that Mr. Arnott should have given the weight of his authority to the almost unlimited employment of local bleeding. He did not regard leeches as very formidable things; but he doubted whether such a profuse employment of them could be beneficial. In children especially he had seen them productive of great injury when so extensively applied.

Mr. Rutherford Alcock said his experience, which had been rather considerable in cases of phlebitis after injuries and amputations, had led him to the conclusion that there could be no general rule laid down for the employment or non-employment of bleeding. There were some cases in which the disease assumed all the characters of an acute and active inflammation, with a full and hard pulse, and high inflammatory fever. In all these he had freely employed bleeding with the most satisfactory results. But there were other cases in which the affection from its very commencement assumed a typhoid character; and in these, of course, no bleeding could be employed. He should think its use nothing less than manslaughter. The patient must in these cases be supported by all possible means. He did not think with Mr. Macilwain that there was any common character in those affected with phlebitis; and if there were, and it could not be defined, he did not know how it could be made subservient to the treatment of the disease. Besides the extreme cases he had mentioned, there were others in which the affection assumed a mixed character; the fever was neither typhoid nor acutely inflammatory, but something between the two. In these he was inclined to think the plan recommended by Mr. Arnott, of copious local bleeding, to be the most beneficial that could be adopted.

Dr. Elliotson said it was plain that in phlebitis, as in most other diseases, there were some cases that must be treated in one way, and others that required just the opposite management. His own experience included only those cases in which the disease had occurred in the course of some medical disorder for which he was in attendance on the patient; as a kind of accident, therefore, to some other disease. In all these he had certainly found the best treatment to be that of supporting the patient; and by this he meant, not that which was commonly confounded with it, the administration of stimulants, brandy, wine, and so on, but the giving of good nutritious food, such as strong beef-tea, eggs, and other things of that class.

Mr. Macilwain would make but one observation in reply to Mr. Alcock. It was said it would be of no use in treatment to know what was the peculiar general condition on which these diseases depended. The subject was too long to be entered on now, and he had given to the world his opinions as to what these general conditions were; but he would give now one example of their application. It was probably generally known and agreed upon what iritis is; that it is an acute inflammation: now when, in cases of iritis, could it be said,—this case depends on suppressed secretion, or other disorder, of the skin, or the kidneys, or some other organ, and when that secretion is restored the iritis will disappear, without either bleeding or any other part of the ordinary treatment; and if these prophecies were repeatedly fulfilled, he thought it was something to learn the real condition of the system on which these diseases depended. Numerous cases of the kind were constantly occurring to him, and he had long invited the attention of the profession to them, in which, by determining what organ was out of order, and by remedying its condition, these local inflammations disappeared as certainly and as speedily as they ever did under the ordinary treatment by bleeding, mercury, and other remedies of that class.—*Trans. of Royal Med. and Chirur. Society, in Lond. Med. Gaz.*

---

*Clinical Lecture, delivered at University College Hospital, February* 9, 1841. By SAMUEL COOPER, Esq., Senior Surgeon to the Hospital, &c.—It is observed by Scarpa, that the femoral hernia seldom occurs in young girls, and still more rarely in men. Yet I have certainly seen many more instances of it in the latter than the former, and am disposed to suspect that the preceding statement, though coming from a high authority, is somewhat questionable. The following is not the only example of femoral hernia that has been noticed in the male patients in this hospital.

*Strangulated femoral hernia in a male subject; operation; gangrene of the bowel; recovery.*

Charles Lutwytch, æt. 51, admitted under Mr. Quain, Jan. 30, 1841, about three o'clock in the afternoon. Twenty-one years ago he perceived a tumor, "smaller than a nut," in his *left* groin; it gradually increased, but gave him no uneasiness, and had never been reducible. About twelve months since, another tumor having formed on the *right* side, the patient procured a double truss.

He describes this last swelling as having always been easily replaced, but liable to descend again whenever the truss was laid aside. At present there is no tumor on this side. Last night he was laboriously employed in carrying sacks of coal in a gas manufactory, and this morning, at 9 o'clock, while he had his truss on, he became very unwell, feeling an inclination to vomit, and to go to stool, with pain in the abdomen and in the tumour of the *left* side, which was found to be larger and harder than usual. He went home, and took an ounce of Epsom salts, which was soon followed by a scanty solid motion.

On his admission, he complained of pain over the whole abdomen, which was much distended, tympanitic, and tender on pressure. He experienced continual nausea, was restless and agitated, and occasionally vomited up a greenish fluid. There was no tumor on the right side; but in the left groin was found an oval, tense, painful swelling about as large as a goose's egg, slightly moveable on its base, which was narrow, and extended transversely. The upper margin of the tumor was concave, and thrown over Poupart's ligament, which could be partially traced beneath it; and, on the other side, the femoral artery was felt pulsating. There was no preternatural heat nor discolouration. The taxis having been tried without success, the patient was placed in a warm bath; and as soon as collapse and faintness had been thus brought on, the taxis was unavailingly tried again.

At 6 o'clock, Mr. Quain, finding that there was no chance of returning the parts by the taxis, recommended the operation; but this proposal was at first declined. The pulse was now fifty-four, and rather small; the belly more tense and tympanitic; the tongue dry, but clean; the vomiting continuing; but the griping pain less severe. On the principles advocated by Dr. O'Beirne, an œsophagus tube was introduced about two feet up the large intestines, but no flatus escaped; and, conseqently, no opportunity was afforded of testing the efficacy of such discharge of air in promoting the success of the taxis.

Passing over some minor circumstances, I will next notice the operation. The hair having been removed, and the integuments held up in a fold, Mr. Quain made an incision across the narrow diameter of the tumor, about three inches long, and directed obliquely inwards, and then another about one inch in length, directed inwards, and joining the first about its centre at a right angle. The sac was then carefully dissected down to, and a piece of it having been lifted up with a pair of forceps, was opened, and the opening enlarged with the aid of a director, whereby a considerable mass of brownish omentum, full of highly congested vessels, was exposed; and on turning this up, some folds of intestine were perceived at the inner side, of a deep and uniform slate colour, and without any visible ramification of vessels. The sac contained no fluid, and the omentum was adherent to the neck of the sac, (a circumstance explaining the irreducible state of the hernia, even from an early period of it;) but the intestine itself was quite free from adhesions. Mr. Quain having reached the stricture, which was a remarkably tight one, introduced Sir Astley Cooper's hernia knife, under the guidance of the left fore-finger, and divided the stricture to the extent of a few lines; but finding this insufficient, he made another slight cut. Both incisions were almost directly inwards. He then began to return the intestine, and, whilst doing so, about half an ounce of thin fæcal matter escaped into the sac; and, on examining the intestine, an opening was detected in its anterior part, at the distance of an inch and a half from the stricture, about four lines in diameter, and the edges of which were very soft, dark coloured, and irregular. Within a few lines of this opening was also noticed a dark spot of nearly the same size, and evidently in a state of gangrene. Mr. Quain next passed a loop of thread through the sound portion of intestine at each side of the opening, and, with some difficulty, reduced the rest of it, leaving the opening itself in the neck of the sac, where it was retained by means of the loop of thread, the ends of which were tied over a fold of lint. The protruded omentum having been cut off, the wound was simply covered with lint. Soon after the patient had been put to bed, the house-surgeon found it necessary to tie two small omental arteries, and, at this time, during a slight effort made by the patient, a gush of thin serous fluid, slightly tinged with blood, took place from the wound. Lint, wetted with cold water was now applied.

January 31st, 1 o'clock A. M.—Occasional discharge of the same kind of fluid from the wound, but no return of bleeding. Patient feels easier; belly is less tense, though tender on pressure; no vomiting. Pulse 60.

℞. Hydr. Chlorid. gr. ij.; Morphiæ Hydr. Chlor. gr. one-eighth; ft. pil. quaque hora sumenda. Poultices to the abdomen.

8 o'clock A. M.—Has slept since 4 o'clock; is now remarkably easy. Tenderness of abdomen diminished; tongue yet dry but clean; no vomiting. Pulse 68. Bed-clothes thoroughly wetted with the thin fluid, slightly tinged with blood, discharged from the wound. Some air escaped from the opening in the bowel, and the griping pains which had been occasionally felt were relieved by it. Same means continued.

1 o'clock, P. M.—Tenderness of abdomen continues.

Ant. Tart. gr. one-fourth, with the calomel, every four hours; and sinapism applied to the abdomen.

10 o'clock P. M.—Discharge of fluid from the wound much lessened; belly rather tympanitic, and tender on pressure; pulse 76; disagreeable smell emitted from the wound, and the lint stained with dirty brown matter; bowels not open. To continue the same plan, with eighteen leeches to the hypogastrium, followed by fomentations and poultice.

February 1st.—Has rested well; a free discharge of fecal matter from the wound; pulse 86.

2d.—Small doses of the hydrarg. cum creta substituted for the pills of calomel, &c.

3d.—Tenderness of the abdomen diminished. A copious evacuation of fecal matter from the rectum; that from the wound lessened. The loop of thread is withdrawn.

*Vespere.*—In the early part of the day five or six loose stools, with griping and tenesmus, and some traces of blood in them.

To discontinue the pulv. hydrarg. c. creta, and have an enema of starch mucilage, with ʒj. Tinct. Opii, and take one-fourth gr. of morphia.

From the wound thick green feces discharged.

4th.—Pulse 60. Tongue furred. Bowels moved three times in the night. Tenesmus continues, with tenderness in the umbilical region. Ordered

℞. Liq. Opii Sedativ. ♏vj.; Aq. Menth. ℥ss. ft. haustus 4tis horis sumend. A sinapism to the epigastrium and fomentations.

*Vespere.*—Bowels still much relaxed. Pulse 70, and compressible.

Omit. Liq. Opii Sedat. and have

℞. Pulv. Cretæ Co. c. Opio, ℈j.; Pulv. Catechu, gr. x. 4tis horis.

5th.—Tenesmus and griping relieved; two or three fecal evacuations in the night principally from the wound. Beef-tea, with wine and milk.

In the evening a considerable discharge of feces from the wound; tènderness of the epigastrium; loss of appetite. Pulse 72, and fuller. Tongue white.

Hirud. xii. ad epigastrium, and omit the powders.

6th.—Feces abundantly discharged from the wound. Patient seems low. No tenesmus, yet frequent inclination to go to stool.

7th.—Generally better. Discharge of feces sometimes from the wound; sometimes from the rectum. Wine with arrowroot.

8th.—A free evacuation this morning per anum. The fecal discharge from the wound lessened.

9th.—Bowels opened three times in the night. Six leeches on epigastrium, for pain in that region. Wound granulating.

10th.—Improving. All tenderness of abdomen had ceased. One regular motion, and less fecal discharge from the wound. A mutton-chop allowed.

14th.—Went on well to this date. A good deal of discharge of feces from the wound today, and parts around excoriated, which were relieved by being smeared with lard, and latterly bathed with a lotion, composed of oxide of zinc diffused in water.

15th.—An aperient clyster administered.

16th.—This produced a copious evacuation from the large intestines, and much general relief to the patient. One mutton chop and two ounces of wine daily.

20th.—No blood with the stools, and less discharge of fecal matter from the wound.

24th.—Vegetables prohibited; wine, beef tea, &c. continued. Going on well.

March 4th.—No fecal discharge from the wound since yesterday evening, and it appears to be healed. Bowels open every day.

5th.—Cicitrization complete, and health good.

*Remarks.*—No doubt this case was originally an omental hernia, and complicated at an early period with adhesion of the omentum to the neck of the sac, so as to account for its always having been, as the man described, irreducible. In his laborious employment at the gas manufactory, on the morning specified, a piece of bowel protruded, and the case changed into a strangulated entero-epiplocele. The femoral hernia, though not common in males, is not so rare as Scarpa supposed, but often overlooked on account of its small size. In men, however, of laborious employments, like our patient, whose work consisted in carrying heavy sacks of coals, and who have been long afflicted with such a rupture, the tumor, if not kept up, (as this seems not to have admitted of being,) will gradually attain a size that must excite the patient's notice; and in the case before us we find that the swelling was, after the addition of the bowel to the contents of the sac, as large as a goose's egg. In women, though the hernia is likewise generally small, examples are to be found in which it occasioned a tumor extending two-thirds of the way down the thigh.

The case at present under consideration at all events cannot fail to make you remember that men are sometimes the subjects of femoral hernia; and that, with reference both to the taxis and the operation with the knife, you will be likely to commit serious and dangerous blunders, unless you make out the true nature of the hernia by careful examination. Now the femoral hernia may always be known by Poupart's ligament being above the neck of the tumor. In an inguinal hernia, the spine of the os pubis is behind and below this part of the neck of the sac; but, in the femoral hernia, it is on the same level, and on the inside of it.

In the particulars of the case before us, Poupart's ligament is mentioned as being perceived to extend over the neck of the tumor, the fundus of which is also described as being thrown up over that ligament, as usually happens when the hernia is of a certain size. The remarkable tightness of the structure—a circumstance particularly common in the femoral hernia of the male subject—was likewise exemplified in the present instance.

The gangrenous and ruptured conditions of the protruded bowel, as discovered when the hernial sac had been laid open, though discouraging, did not, as you have seen, absolutely preclude the possibility of a favourable termination. Here the adherent and diseased portion of the omentum was removed; but the partially gangrenous bowel, which had burst, not being adherent, and having but a limited opening in it, was reduced, and the burst portion of it kept in the neck of the sac by means of the thread tied over a roll of lint. This was done that the fecal matter might escape outwards, and not pass into the cavity of the peritoneum. Had the bowel been fixed by adhesions to the neck of the sac, then, of course, no ligature would have been necessary or proper.

The copious discharge of serous fluid from the wound, as related, came, as I need hardly say, from the peritoneal cavity. I have witnessed several instances of the same occurrence.

One point deserving notice in this case was the mildness of the constitutional disturbance, notwithstanding the complication of the hernia with gangrene and rupture of the intestine.

The case also adds another instance to several which have been seen in this hospital, where, under strict and persevering antiphlogistic treatment, and especially under judicious diet, and with proper attention to cleanliness, nature not only saves the patient, under the disadvantages which this man experienced, but prevents the continuance of that loathsome affliction, an artificial anus.

The discharge of serum, and the tenderness of the belly, were regarded as indications of a tendency to inflammation in the peritoneum, and the calomel and other means prescribed were intended to control any increased action in that membrane. The calomel and tartarized antimony, however, were discontinued when signs of gastric irritation began; or when the mucous, and not the serous membrane, appeared to be the seat of disorder.—*London Medical Gazette.*

---

*Two cases illustrating the effects of Contre-Coup on the Brain.*

*To the Editor of the Medical Gazette.*—The two following cases of injury of the head, which occurred within a short interval of each other, appear deserving of being placed on record, chiefly on account of what the *post mortem* examinations disclosed. The appearances found on dissection in both cases, show how the same impulse, which, on the principle of *contre-coup*, frequently causes fracture of the skull, in severe injuries of the head, to take place at the point opposite to where the blow was received, may produce a fatal bruising of the substance of the brain at the part opposite to the seat of the blow, even when the injury has not been so great as to cause fracture of the bone at the situation referred to; or, as one of the cases shows, when there has been no fracture of the skull at all. Some writers, in treating of *contre-coup*, and showing the application of that principle to "concussion" of the brain,* have adverted to the effects thus produced on distant parts of the brain by blows on the skull, independently of fracture: but I have not, in my reading, met with any cases that illustrate the results of this kind of injury in so striking a manner as those which I now beg to send you.

I am, sir, your obedient servant,

ALEXANDER SHAW.

CASE I.—A. B. æt. 28. This man was admitted into the Middlesex Hospital, Feb. 8th, in a comatose state, having two bruised and open wounds of the scalp, and it being reported that he had fallen, on the previous evening, from a considerable height. The wounds, which were near each other, were situated on the right side of the head, over the prominence of the parietal bone, and between that part and the sagittal suture. In one of the wounds the bone was denuded to an extent about the size of a sixpence. The patient could be easily roused from his stupor; and was then fretful. He was frequently sick and vomited. His pupils dilated and contracted in obedience to the light. His breathing was tranquil.

For the first six days the treatment corresponded with what is usually adopted in cases of supposed concussion of the brain: he was first freely purged with calomel and jalap; was bled from the arm, and afterwards cupped from the temples; had cold lotions applied to his head, and took calomel and antimony in small repeated doses, to the extent of making his gums sore. During this period he appeared to be progressively amending; he became more sensible, lying for a considerable part of the day awake, as if observing what occurred in the ward; answering questions at times to his friends or the nurse; and getting out of bed to void his urine or use the close-stool. The wounds of the scalp assumed an improved appearance and discharged healthy pus.

On the evening of the 14th, shortly after being so well as to answer a question put to him by the nurse, he was seized with a fit resembling an epileptic attack. For this he was bled from the arm by the house-surgeon; and a full dose of calomel and jalap was administered.

15th.—He has had several more fits. They consist of a spasm and convulsion confined to the right side of the body. Each fit commences by the head being drawn to the right side, the eyes being directed to the same side, and remaining fixed, as long as the fit lasts, in that position; the muscles of the right half of the body now become rigid, and presently the arm is alternately bent and extended, while the patient draws his breath in a succession of short rapid inspirations, accompanied with a crying sound. His stupor is increased; yet the breathing remains soft and tranquil, and both pupils obey the stimulus of light in an equal manner. The pulse varies in rapidity and force at different times.

16th.—There is not much alteration since yesterday. At first the fits were severe, but occurred only about once in every six hours; now they return frequently, and are of short duration. The calomel and jalap have been repeated, and a large blister has been applied to the nape of his neck.

Evening.—It is now observed that the right side of the body, besides being affected with the spasms above described, is paralysed. Yet he preserves sensation on that side. When pinched on the right side, he manifests distinct signs of pain; but it is by moving the left arm, not the arm that is pinched. His face being flushed, and the skin hot and dry, and the pulse hard, he was bled from the temporal artery to the extent of sixteen ounces.

17th, morning.—He is no better. A consultation having been called, it was resolved to remove with the trephine the portion of bone denuded in the upper part of the wound. This was done; but the dura mater underneath presented no unnatural appearance. The fits continued during the day as frequent and as severe as before. He was ordered a quarter of a

---

* See a Clinical Lecture by Sir Charles Bell, Medical Gazette, vol. viii.

grain of tartar emetic in water, every two hours.

18.—There is no observable change in regard to the convulsions, except that they perhaps succeed each other more frequently than before, being sometimes three in the hour.

19th.—Early this morning he died.

*Post-mortem Examination.*—Upon removing the skull-cap, the dura mater presented a natural appearance, except at the point corresponding with the trephined hole in the skull, where some granulations had risen. When the dura mater was reflected, so as to expose the brain, an effusion of blood was found extending over the lateral part of the left hemisphere: but upon the right hemisphere there was no similar effusion, or any morbid change. The effusion of blood on the left hemisphere formed a thin layer, spread equally over the surface, except at the temple, where the clot was more abundant. On examining this part, (which, following an oblique line from above downwards, was to be accounted the point opposite to where the blow was received) a considerable portion of the brain was found to be broken down to a soft pulp: and from this part it was obvious the blood effused around, and which extended likewise towards the base of the brain, had proceeded. The particular portions of the brain which had suffered this bruising of their texture to the greatest degree, were the anterior part of the middle lobe, which lies in the sulcus formed by the greater *ala* of the sphenoid bone; and the posterior edge of the anterior lobe, at the most external parts. No fracture could be perceived in any part of the skull.

Case II.—A strongly built, muscular man, was admitted, at one o'clock P. M., March 21st, in the hospital, being in a state of insensibility, consequent, as it was reported, on a fall from a ladder. It was stated by his friends, that on the previous evening, while standing on a ladder about eleven feet from the ground, he fell backwards and lighted on his head. He immediately became insensible, and has remained in nearly the same condition till the present time. Last night he was visited by a surgeon, who bled him at the arm.

Upon examining the back of his head, a bruised wound of the scalp was found, situated over the tubercle of the occipital bone. The patient, although comatose, could be roused, when stirred and loudly spoken to, so as to open his eyes and put out his tongue. He slightly resisted, with both eyes, having the eyelids opened with the fingers. It was ascertained that the pupils were contracted. He was observed to move his arms and legs. His pulse was soft, and at 65: his breathing was noiseless and without labor. He was ordered a powder consisting of

Jalap, grs. xx., and Calomel, grs. v. To have his head shaved, and a cold lotion applied.

Upon being visited in the evening, his face was found flushed, with drops of perspiration on it; and the pulse was above 100, hard and full. He was ordered

V. S. ad. ℥xvi.; and to commence taking Calomel, grs. ii., with Tartar Emetic, gr. ¼ every four hours.

22d, 3 o'clock A. M.—I was summoned to the hospital in the night, under an alarm that the patient was dying. It appeared at the time that the attack had proceeded from mucus accumulated in the air-passages, and the patient had not strength to expectorate. He recovered after the house-surgeon had swept with his finger some thick mucus from the back of his throat. It was subsequently noticed that in his breathing, although it appeared easy, the throax was remarkably fixed, so that its motion could scarcely be observed; and the attempts that he made to cough were feeble and imperfect. During this day his stupor appeared more profound. He allowed his arms to fall when raised, as if they were paralytic. His legs are never moved. His urine and fæces pass from him involuntarily. The pupils are still contracted. As his head was hot, and his pulse rather full, twelve leeches were ordered to be applied to his temples, and afterwards a bladder of ice to be kept to his head. The calomel and antimony are continued.

23d.—At five this morning he died.

*Dissection.*—Some ecchymosis was found at the back part of his head, where he fell. Upon removing the skull-cap, the dura mater presented its natural appearance. When this membrane was reflected, a layer of blood was found to be effused over the anterior parts of the right and left hemispheres of the brain, being contained between the dura mater and arachnoid membrane. This blood was most abundant where the anterior lobes lodge on the orbital plates of the frontal bone. Here the brain was extensively bruised, and converted into a substance consisting of coagulated blood mixed with brain. The anterior lobes being sliced down, the injury was found, especially on the right side, to have reached very deeply; so that a communication existed between the broken-down part and the anterior cornu of the right lateral ventricle; and a considerable quantity of blood, obviously derived from the ruptured vessels of the bruised part, was extravasated into this ventricle, and extended likewise into the left ventricle. At the posterior part, where the blow had been received, no marks of injury to the substance of the brain could be perceived; on the contrary, both cerebrum and cerebellum presented a sound appearance at this part. Nevertheless, a fissure of the skull existed here. This fissure began below the tubercle of the occipital bone, and was traced on the left side of the perpendicular ridge, to the foramen magnum. Although a careful examination was made, no injury of the skull could be detected in front, where the brain was so extensively destroyed.

*An Account of Two Cases of Imperforate Hymen.* By Sir B. C. Brodie, Bart., F. R. S.—The author was induced to give to the society the narrative of the cases in question, not so much in consequence of any thing unusual in the cases themselves, as from a wish to awaken attention to the differences between those instances of true imperforation of the hymen, and such as are usually described as belonging to the same category, but which are, in reality, nothing more than cases of congenital closure of the vagina, or accidental adhesion of the walls of that canal.

Dr. Merriman believed that cases of imperforate hymen were of rare occurrence. Two cases had occurred to him in which this kind of obstruction in the vagina had prevented the discharge of the catamenia. The first case was that of a girl, who was under the care of the late Mr. Chevalier, and who supposed that she had closure of the vagina from the occurrence of previous inflammatory action in that part. On examination, however, he (Dr. M.) found that the obstruction was really dependent upon an imperforate hymen, and an operation for its relief was proposed. This was performed by Mr. Chevalier, who merely made a crucial incision through the membrane. The girl eventually did well; but in this case, as in one of a similar kind, related by Dr. Denman, inflammation of the peritonæum came on, and required active depletory treatment.

The other case of imperforate hymen he had seen with Sir C. Bell. In this case the woman was five-and-twenty years of age; and although she had been married for five years, neither she nor her husband had any idea that the hymen was unbroken. It was in consequence of her becoming enlarged, and supposing herself to be pregnant that she applied for advice. Sir C. Bell divided the membrane, and gave exit to two or three pints of catamenial fluid, resembling tar, which had collected in the uterus and vagina, and caused enlargement of the abdomen. It was two or three days before the whole of the matter had come away. He saw this patient more frequently than he did the first one; and he had found, at the end of six or seven weeks from the operation, that the vagina was still so distended as to be capable of holding the head of a child. It was a long time before it contracted to a natural size. Eventually, however, she became pregnant, was delivered of a living child, and did exceedingly well.

He had lately seen a case which bore a great resemblance to the second one related in the paper. In this, it was supposed that the young woman had imperforate hymen. On examination, however, a minute perforation, capable of admitting a probe, and through which a small quantity of catamenial fluid was discharged, was discovered. This Dr. Merriman enlarged, first by the introduction of a probe, and then by the use of bougies, gradually increased in size. Dilatation of a sufficient extent was thus procured, without the loss of the hymen. On one occasion he had found the hymen unruptured during labour. The pressure of the head of the child rendered any surgical division of the membrane unnecessary.

Dr. Moore recollected a case of a young woman, eighteen years of age, who had all the usual indications of menstruation, without any discharge from the vagina. On examination the hymen was found to be imperforate. A trochar was pushed through it, and the catamenial fluid discharged. The orifice remained open. He had seen a case in which the sides of the vagina had become agglutinated, in a woman of twenty-two years of age; in this case the parts were divided by the bistoury, and the patient did well. Dr. Alcock had informed him of an interesting case of a young woman who had suddenly become moribund, and died in two or three hours. She was between eighteen and nineteen years of age, and had never menstruated. There were all the usual accompaniments, however, of that state, such as the enlargement of the mammæ, &c. She had been under treatment for twelve months for a tumour in the pelvic region, which had gradually increased in size, so that at last she could not stand erect. One day she felt something suddenly give way in the abdomen, and felt instant relief; inflammation, however, came on, and she died. On examination, it was found that one of the Fallopian tubes had given way, and the abdomen was filled with a large quantity of chocolate coloured fluid. The uterus was large and flaccid, the Fallopian tubes distended, and the right one ruptured. About two inches of the vagina, near to its orifice, was quite consolidated.

Dr. Ashwell had been rather surprised to hear Dr. Merriman, with so extensive and lengthened an experience, say that he had only seen two cases of imperforate vagina. Under his, Dr. A.'s, more limited observation, four cases of imperforated hymen had occurred. In these all were discovered after puberty; all were operated upon, and did well eventually; although in two of the cases severe peritoneal inflammation supervened, through which the patients were carried with difficulty. Regarding closure of the vagina, it was more difficult to keep this open after operation, than it was an imperforate vagina. In a case of the first kind, in which an incision was made through the agglutinated parts by Mr. Key, and a large quantity of menstrual fluid discharged, the patient remained in Guy's Hospital for two or three weeks, went out, and returned at the end of six or seven months, the vagina having again become closed, and a large quantity of catamenial fluid collected behind the obstruction. A second operation, similar to the first, was performed; but peritoneal inflammation came on, under which the patient sunk in forty-eight hours. In a case of imperforate hymen, discovered for the first time during labour, the membrane was divided by operation, and the patient did well. He, Dr. Ashwell, thought cases of imperforate hymen were not very uncommon.

Sir B. Brodie considered, from the cases related by fellows, that an imperforate state of the hymen was not so rare as he had previously supposed. Dr. Blundell had, however, seen only one case at all similar to the first related in the paper, and Sir C. Clarke not even one. Four cases of closure of the vagina had come under his, Sir B. B.'s, care. In one case a trochar was employed, and a considerable quantity of menstrual fluid disharged; but the patient nearly perished from peritoneal inflammation, and the opening was kept patent with difficulty. In another case, in which the trochar was also used, this difficulty was experienced. In another case, in which the patient died, a considerable quantity of catamenial fluid was found in the abdomen, to which it was supposed to have passed through the Fallopian tubes, as the uterus was not ruptured.

Dr. Elliotson recollected one case of imperforate vagina in St. Thomas's Hospital. He had also a case under his care, in that hospital, of a married woman, who had no vagina whatever, and yet she and her husband had not found out the malformation. Mr. Cline made an incision in the place where the vagina should have been, on two or three occasions, each time going deeper, but nothing came. Of course, the woman had never menstruated.—*London Lancet.*

---

*Case of simple dislocation forwards of the heads of the Tibia and Fibula.* By HERBERT MAYO.—Enright, aged 65, of a spare habit, but hale and ruddy complexion, by employment a stone mason's labourer, was stooping to clear away some rubbish beneath a portico that was just completed, when the stone forming the architrave, weighing towards seven hundred weight, broke in two, and falling upon him bore him to the ground. His right hand was jammed under one fragment, which it was necessary to lift to extricate it. He could not stand for the shock, and some injury to the left knee. The accident happened about half-past five P. M., on the 23d of February; he was shortly after brought to the Middlesex Hospital. I saw him between six and seven.

The first impression, on looking at the injured knee, was that the femur was broken immediately above the condyles, where there was a considerable depression, and there seemed to be motion. But upon a closer examination the femur proved to be entire, the depression being caused by the head of the tibia overlaying that bone. The condyles of the femur, the outer stretching the skin very tensely, were to be felt behind the upper ends of the tibia and fibula, the extremity of the condyles being fully four inches below the level of the articular surface of the tibia. The position which the displaced bones assumed was that of slight flexion. The pulsation of the anterior tibial artery on the instep could not be felt.

Two brief and ineffectual attempts were made by hand to reduce the dislocation, keeping the bones slightly flexed. Then a round towel was passed within the limb, to get a purchase upon the ischium and pubes; this was fastened to the irons of the head of the bed.—Another round towel was secured by a clove hitch upon the malleoli and instep. Using these means, direct extension was made upon the leg, and in about a minute the reduction of the dislocation was accomplished. The limb was then laid straight upon a pillow, and supported laterally by junks.

Upon examining the hand, finding three of the fingers crushed, the flesh of the thumb deeply lacerated, and the skin torn off the whole dorsal surface, I amputated it above the wrist. The patient's progress has been in every way favourable. Some swelling and pain there was of the knee-joint forty-eight hours after the accident, for which twenty leeches were applied; but there is no pain now unless the joint is moved; and the swelling has disappeared.

This is the only case of simple dislocation of the head of the tibia forwards that I have witnessed. I saw, some years ago, with Mr. Andrews, of Stanmore, a young gentleman who, a few days before, had suffered simple dislocation of the head of the tibia backwards, which Mr. Andrews had reduced. He had used direct extension, and had found considerable force necessary. The accident had happened in the following manner. The young gentleman was riding a pony on the common, and meaning to pass a tree that stood in his way on the one side, when the pony swerved, and carried him by on the other side of the tree, which took the tibia, and forced it backwards, dislocating it behind the femur.

In the case which I have narrated, it is to be supposed that when the unfortunate man was borne down by the mass of stone, his leg, with the knee straight must have been stretched obliquely forwards, and the foot have been fixed against some object, when the strain falling on the crucial ligatures, tore them through, when the condyles of the femur would have been forcibly driven behind the tibia and fibula.

I am disposed to mention, as connected with the subject of dislocation, a simple contrivance, perfecting the commonest apparatus for reducing dislocations of the hip, which I have recently had put up at the Middlesex Hospital.

There was in the room destined for this purpose a ring let into the floor; and opposite to it at twenty feet distance, was an upright iron bar, fixed by its ends into the wall, having catches at different heights adapted to the hook of a set of pullies. The patient being laid upon a common iron bedstead placed lengthways between the ring and the vertical bar, the two latter furnished points of attachment for making horizontal extension of the thigh. What I have added is an horizontal bar running below the ceiling of the room, the ends being attached to the joints of the floor below. This bar is parallel to a line adjoining the ring and vertical bar just described. It therefore allows the hook of another set of pullies to hold to it, and so affords the means of making a strain upon the dislocated femur, transverse to the axis of the limb, simultaneously with the longitudinal strain.

---

*Case of Aneurism of the Aorta, by* JOHN GAY, *Surgeon to the Royal Free Hospital, &c.*—John Scott, ætat 28.—A little man, with red hair, and other indications of a scrofulous constitution, was admitted into the Royal Free Hospital on the 25th of March. He stated that he had worked for some years at Liverpool, as an anchor wright, but that at Christmas he was thrown out of employment; he was obliged, consequently, to wander about, in search of work, during the most inclement part of the winter, barely clad, and as badly fed; and to the sufferings which he thus endured, from cold and want, he attributes his present complaints. About three weeks before he arrived in London he perceived the first symptoms of indisposition, which consisted in an almost frost-bitten state of the lower extremities, cough, and slight dyspnœa, succeeded by uneasiness about the chest, expectoration tinged with blood, and occasional epistaxis. These symptoms increased, and, shortly afterwards, a small tumor (without pain) made its appearance on the right side of the sternum. In this state of extreme wretchedness he applyed for admission at the Asylum for the Houseless Poor, and was thence sent to the Royal Free Hospital; he was there put into a warm bath, and I saw him immediately after.

March 25.—After a bath, skin hot and perspiring; lips purple; breathes with difficulty, and expectorates a consiederable quantity of mucus, tinged with blood; pulse 110, small, but with a slight jerk; unable to lie on the left side, a posture which gives him severe pain about the right hypochondriac region; tongue rather white; no appetite.

On the right of the sternum, and between the cartilages of the first and second ribs, is a pulsating tumor, about the size of a walnut; the skin covering it is slightly inflamed; yields on pressure, as though fluid were confined beneath, and appears to be very thin; if it be pressed but ever so gently, the patient complains of severe pain extending itself towards the right axilla. The pulsation in the tumor is both heard and felt to be the same precisely as that of the heart, and without the bruissement that aneurismal tumors generally yield; the sounds of the heart itself are heard more distictly, and its impulse is more violent on the right than on the left side of the sternum, and most especially on the apex of the tumor; the arteries of the wrists have no sensible difference in their action; and the same is observable of the carotids. There is, however, a considerable retrocession of blood into the right jugular, synchronous with each ventricular systole.

The respiratory murmur is louder on the left side of the chest than on the right, where it is probably obscured by some large crepitation, which is diffused over the whole of that side, but only to be heard behind. Owing to the embarrassed condition of his breathing, and the pain which he experiences from any movement, I am not able to obtain any further auscultatory evidences. I ordered twenty leeches to be applied to the chest, and prescribed the following medicine:

℞ Tincture of digitalis, M 12;
  Antimonial wine, ʒss;
  Solution of acetate of ammonia, ℥ss;
  Sulphate of magnesia, ʒj;
  Camphor mixture, ℥j.

Mix this for a draught, to be taken every four hours.

26. Has passed a comfortable night; cough not so violent, and the expectoration presents no traces of blood, consisting chiefly of frothy mucus; bowels relieved; pulse still jerking slightly. Twelve leeches to be applied to the chest; the draughts to be continued.

27. Skin hot; perspired freely during the night; rest disturbed by unpleasant dreams; felt much relief from the bleeding; other symptoms more favorable; pulse 96, moderate.

℞. Antimonial wine, ʒss;
  Tincture of digitalis, M 12;
  Compound tincture of camphor, M xxv;
  Water, ℥j.

To be taken every four hours; and three grains of mercury, with chalk, at bed time.

30. Has continued his remedies, with much relief; but this morning he complains of nausea and vertigo; pulse 90, and feeble. Continue the draughts without the antimonial wine.

April 2. Expresses himself as being "very comfortable;" but the tumour has become more prominent, and larger at the base.

On exploring the chest to-day, I find that the sounds of the heart are heard, and its impulse felt more forcibly on the site of the tumour than on any other part of the chest. A slight bruissement accompanied the first sound, which is distinguishable *only* over the tumour; respiratory sounds, louder on the left side than on the right, and accompanied with some rhonchus and sybilis; very little of the moist crepitus remaining; percussion gives a dull sound over the whole of the left side; this test could not be applied to the right, on account of the uneasiness to which it would have subjected him. Continue the draughts, with twenty minims of antimonial wine; meat diet, without porter or wine.

13. Since last report, the tumour has increased considerably, and acquired a somewhat irregular surface; breathing has become perfectly tranquil whilst he remains in a state of rest, and his cough troubles him but very slightly; sleeps well at night, but cannot lie on the left side; in any other posture, with ease; gentle pressure on the tumour gives pain; and disturbs the tranquillity of his respiration. For the last five or six days he has again complained of giddiness and tendency to faintness, and his pulse has become feeble and irregular, beating about 96 times in the minute. The abnormal sounds have quite left the chest, as far as respiration is concerned, but the vesicular murmur is still more clearly discernible on the left than on the right side; the skin covering the tumour has recovered its healthy appearance. A lotion was kept applied to the tumour, consisting of nitrate of potass and muriate of ammonia, of each ʒij, to half a pint of water.

27. For the last three days he has complained of headach, and to-day has been sick several times. On examining the chest, I find (what I have not before noticed) a bulging of the left side of the chest, chiefly about the curvatures of the fifth, six, and seventh ribs; no uneasiness in this region, but a dulness on percussion; in other respects as at last note.

At the suggestion of an esteemed friend, I have directed him to take the following, instead of the draughts, which appear to have kept his circulation at a very low ebb:—

℞ Tincture of muriate of iron ♏ 12;

Cinnamon water, ℥j.

To be taken every four hours.

May 8. Complains of uneasiness in the region of the tumour, which he has felt for some days, but which, to-day, amounts to pain; pulse 105, with more power; skin rather hot and feverish. Twelve leeches to be applied to the tumour, and a saline mixture, with sulphate of magnesia, to be taken every four hours.

9. Bowels well relieved; pain and excitement subdued.

13. Pain gone; complains of uneasiness in the bowels; pulse 100, moderate.

℞ Tincture of digitalis, ♏ 20;

Aromatic confection, ʒss;

Peppermint water, ℥iss.

To be taken every four hours.

27. The action of the heart and arteries has again become feeble and irregular; and since this state of the circulation has been again obtained, the tumor has become decidedly lessened in bulk; to-day I have been able to sound the right side of the chest; the degree of resonance is very feeble, and offers a striking contrast with that which the whole of the right side yields behind; respiratory murmur heard more clearly in every part of the chest, excepting around the tumor, but generally more loud on the left than on the right side; the voice reverberates considerably on the right side, and approaches in its character towards œgophony on the same side, behind, may be detected a bruit, accompanying the first sound of the heart.

In other respects he appears tolerably well; cough has entirely ceased, and he is enabled to sleep well; pulse weak and irregular, 100.

June 8. Better.

℞ Tincture of digitalis, ʒviss;

Tincture of hyoscyamus, ʒiss.

Thirty-five drops to be taken every six hours.

12. The tumor is nearly reduced to a level with the parietes of the chest, which in that situation have become sensibly thicker; pulse accompanied with a slight jerk. Increase the number of the drops to 45, three times in the day.

15. Impulse of the heart slightly increased; first sound of the heart prolonged, and accompanied with a bruit, which can be heard most distinctly by applying the stethoscope rather above, and about half an inch to the left of the tumour; pulse 95, firm, but intermitting. Increase the drops to 50, three times a day.

July 3. The tumour has been gradually receding, and is no longer observable; the pulsation, however, is felt on laying the hand over the site which it occupied.

8. To-day the man left the hospital, without my knowledge; his breathing having become perfectly still, and every symptom so thoroughly alleviated, that he was sure he could return to work, if he could obtain it; upon this pretext he left.

The foregoing extracts, from my note-book, include *all* the important points, which I was enabled to glean from an almost daily examination of this individual's case. When he came under my notice, I was led to consider the indictaions which the history and aspect of his disease appeared to suggest; and, with the aid of pathology, most valuable even in its yet infantile stage, to make them the grounds of a plan of treatment, to be rigorously pursued. It had been my lot to see many instances in which the plan of Valsalva (now practically advocated) was pursued; and, from its repeated failures, and only occasional (and that apparently haphazard) success; I resolved to reflect, ere I gave a patient with aneurism the chances of an alternative, but little if aught better than those of its spontaneous termination. But it is not my intention to speak disparagingly of the individual, to whose treatment of aneurism I have alluded. Much credit is due, as it has been awarded, to that great man, for his courageous and happy conflicts with disease; but it is probably a matter of doubt, whether it is wise now, with the present condition of society, to imitate him in any thing but the intellectuality and profound knowledge which presided over all his procedures. The searing iron and boiling oil, which were comparatively innocuous to our more hardy ancestors, have been superseded by more mild and compatible surgery; not only as the result of that progress which surgery, in common with all other scientific discovery, has made, but as demanded by those modified conditions of the human organization which have been brought about by the usages of an advanced era in the history of human society: so that (in my humble opinion) the arguments for the adoption of a certain plan of treatment, in any disease, are not established by the facts, however abundant, of its having been followed with eminent success, some century or more since. And, to my mind, this is the only way to solve a discrepancy which, unaccounted for, would render the observations of the most gifted members of our profession but of little estimation, either for the establishment of a theory, or as rules of practice. Thus, whilst Valsalva's treatment of aneurism was supported by the physicians of his own time, and has been since, by Guerin, Pelletan, Sabatier, and others, it has been deemed useless by Boyer, Roux, Cooper, and other masters of modern surgery.

But, besides, the blood contains the material which is to repair the injury which an aneurismal artery has suffered. It has been said by eminent pathologists, that the fibrinous lamellæ contained in the sac are secreted by it; but this, I think, is not borne out by a careful examination of the concretion; at all events, if not *directly*, *it must indirectly* be a separation from the circulating fluid, and consists principally of fibrin. Now the tendency of repeated venesection is to diminish very considerably this element of the blood, and with it the disposition in that fluid to deposit a coagulum, to say nothing of the serious consequences of procured anæmia on the system at large. Hence this course does not, *à priori*, appear calculated to lead to a spontaneous cure of aneurism, but rather, on the other hand, to weaken our hope of such a termination, inasmuch as it wastes the *material*, and the *power* by which it is applied to the reparation of the diseased and enfeebled artery. But can a *remora* of the circulation be induced without such a sacrifice as blood-letting necessarily involves? Can the blood be made to circulate in a preternaturally languid current, and retain, at the same time, its normal quantity and proportion of ingredients? These questions I attempted to solve by the treatment of the foregoing case, and from the results am induced to think that the course pursued is strictly in harmony with what we know of the nature of aneurismal tumours. Throughout, this individual was kept (with the exception of a short period, when the treatment was reversed) in a state bordering upon syncope, taking care not to go so far as to produce vomiting. If symptoms of bronchitis or acute pain in the region of the tumour manifested themselves, leeches were applied, and gave very marked relief; at the same time, nutriment was given to him in the form of animal broths as frequently as he was disposed to take it, and attention paid to the state of his bowels.

The situation of the aneurism in this case is a question upon which I will not venture an opinion; but I felt assured in my own mind that reparation of a permanent character had taken place. My object, in the recital of this case, will be answered, should it tend to elicit facts, either confirmatory of, or hostile to, the views which led me to adopt this (not novel, but unadvocated,) plan of treatment.—*London Lancet.*

